# The significance of the left atrial volume index in prediction of atrial fibrillation recurrence after electrical cardioversion

**DOI:** 10.15171/jcvtr.2017.08

**Published:** 2017-03-18

**Authors:** Mehrnoush Toufan, Babak Kazemi, Negin Molazadeh

**Affiliations:** Cardiovascular Research Center, Tabriz University of Medical Sciences, Tabriz, Iran

**Keywords:** Atrial Fibrillation, Cardioversion, Left Atrial Volume Index

## Abstract

***Introduction:*** Electrical cardioversion (ECV) is a safe method for the treatment of atrial fibrillation. It seems that left atrial volume index (LAVI) could be a good marker in predicting the success of ECV. The purpose of this study is to assess of the significance of LAVI measurement before ECV in predicting the recurrence of the AF.

***Methods:*** Fifty-one patients with AF, selected for ECV were studied in the cardiology department of Tabriz University of medical sciences. The clinical and demographic data of all the patients were obtained. Echocardiography was performed before and also three months after ECV. Patients were separated into two groups: those who maintained SR and those with relapse of AF diagnosed by clinical manifestations and electrocardiography (ECG).

***Results:*** Sinus rhythm (SR) was maintained in 76.5 percent of the patients following the three months after ECV. The age, sex and the body mass index (BMI) were not significantly different between SR and AF groups. Two groups showed no significant differences considering pre-ECV medical history including medications and systemic diseases. The initial LAVI of SR group was 42.21±12.4 mL/m^2^ and AF group was 96.08±52.21 mL/m^2^, the initial LAVI was significantly different between two groups (*P * = 0.000). The LAVI of SR group decreased significantly (5.69±0.74 mL/m^2^) after three months, LAVI decreased from 42.21 ± 12.4 ml/m^2^ to 37.51 ± 10.52 mL/m^2^. (*P * = 0.000). The cut-off point of LAVI value in predicting the maintenance of SR was 55 mL/m^2^.

***Conclusion:*** The present study indicates that LAVI is a powerful forecaster of the recurrence of AF after ECV. The LAVI measurement could be a useful method in the selection of the patients with AF for ECV.

## Introduction

The prevalence of atrial fibrillation (AF) is approximately 1.5%-2% of the general population and is the most common sustained cardiac arrhythmia. The general treatment strategy of persistent AF is to restore and maintain the sinus rhythm (SR).^1-4^ Electrical cardioversion (ECV) is a simple and safe method to restore the SR. Previous studies reported the initial success of ECV as 50%-90%. However, recurrence of AF occurred in almost 60% within 3 to 6 months, especially in the first 2 months.^5^ Many factors could affect and predict recurrence of AF after ECV and better treatment strategies may be planned by understanding them. Previous studies have reported various potential predictors such as the age, underlying cardiovascular diseases and related risk factors, duration of AF, inflammatory markers and the size of the left atrium.^2,6-8^

The relationship between the size of the left atrium and the AF recurrence has been showed in many investigations. Many of them reported this link using the left atrial diameter (mostly the anteroposterior dimension).^9-11^ However, this measurement may not reflect the complex changes in the size of left atrium. In a few number of studies, the left atrial volume index (LAVI) has been shown to be stronger than the anteroposterior left atrial dimension in predicting the recurrence of the AF.^12,13^ The purpose of this study is to assess of the significance of LAVI measurement before ECV in predicting the recurrence of the AF.

## Material and Methods

### Study design

This study was planned as a prospective cohort of patients with AF rhythm.

### Study population

Seventy patients over 18 years old, who were admitted to the Cardiology Center of Tabriz University of Medical Sciences and diagnosed with non-valvular persistent AF, were enrolled in the study (2013-2015). All the patients were requested to sign a written informed consent. The ethics committee of Tabriz University of Medical Sciences approved the study. The patient history, physical examination and electrocardiography (ECG) findings formed the diagnosis; and based on that the patients were selected for ECV procedure. Exclusion criteria were history of other atrial arrhythmias (including paroxysmal AF), acute coronary syndrome, congenital heart disease, severe valvular heart disease, mechanical or bio-prosthetic heart valves and permanent pacemaker, patients in which a thrombus was detected in the left atrium, thyroid dysfunction and finally history of cardiac surgery.

### Clinical examinations

Clinical data including age, sex, body mass index (BMI), hypertension, diabetes mellitus, coronary artery disease, medications and duration of the AF rhythm were recorded before the echocardiographic evaluation.

### Echocardiographic Data

Two-dimensional transthoracic (TTE) and transesophageal (TEE) echocardiographic examinations were done before and three months after ECV with the Vivid 7 (Vivid 7, GE Ultrasound, Horten, Norway) ultrasound device. Two-dimensional (2D) imaging (using apical four-chamber and apical two-chamber views), M-Mode and Doppler echocardiographic techniques were performed by an experienced cardiologist (blinded to the patients’ medical history). All patients were examined according to the guidelines of American Society of Echocardiography. The left ventricle wall thickness, systolic and diastolic ventricular dimension, ejection fraction, left atrial dimension were measured. The LAVI was achieved using the biplane area length method. The area in the apical four-chamber view (A1) (not taking the initiation of pulmonary veins and left atrial appendage into account), two-chamber view (A2) (after detection of teapot sign for accuracy)^14^ and the smallest long axis length of the left atrium at ventricular end-systole were measured. Then the LAVI was calculated by this formula: (0.85A1*A2)/L. The correction for BSA was applied on LAVI.

### Electrical cardioversion

In our study, the ECV procedure was performed by an experienced cardiologist blinded to the patients’ history. Sedative medications (Midazolam 1.5 mg IV) were administered to all the patients before the cardioversion. Shocks were delivered using a bi-phasic defibrillator (Lifepak 20e defibrillator/monitor, Physio-Control, Inc., Redmond, USA). Paddles were put on the second right intercostal space and the left side of the mid-axillary line. External bi-phasic DC shocks were started with 100 Joules (J) and followed by 200 J and 300 J in the case of failure in generating the SR. Those patients who restored SR and maintained it for 24 hours were included in the follow-up.

### Clinical follow-up

Patients with maintained SR were prescribed Warfarin (5 mg, orally) for 6 weeks in order to achieve an INR of 2 to 3 and the antiarrhythmic therapy (Tab Amiodarone 200 mg BD in 15, Tab Propafenone 150 mg BD in 21, and Tab Felecainide 100 mg BD in 15 patients) to prevent the recurrence of AF Patients were separated into two groups: those who maintained SR and those with relapse of AF diagnosed by clinical manifestations and ECG (SR and AF groups). Clinical and echocardiographic examinations were done again at this point.

### Statistical analysis

Descriptive statistics were performed. Normality was tested by Kolmogorov-Smirnov and group comparisons were performed using chi-square and Mann-Whitney U-test.

Multiple logistic regression analyses were employed to find out the factors affecting the ECV success and the recurrence risk of the AF. Receiver operating characteristic (ROC) analysis were produced to evaluate LAVI as a predictor of maintenance of SR after ECV and to determine an appropriate cutoff point for LAVI for the prediction of AF recurrence, according to sensitivity and specificity. All tests are two-sided, and P-value less than 0.05 were considered to be significant. Statistical analysis was performed using SPSS 18 (SPSS Inc., Chicago, USA).

## Results

Nine patients were dropped out of study because of the detection of exclusion criteria during the study. Four patients were not included in the post-ECV follow-up for failure in maintaining SR and six people were dropped out of study for personal reasons. Among the remaining fifty-one patients, the mean age was 58±12 (21 to 80) and 52.9% (n = 27) were male. SR was maintained in 76.5 percent (n = 39) of the patients, whereas the AF reoccurred in 23.5% (n = 12). The age (*P* = 0.657), sex (*P* = 0.276) and the BMI (*P* = 0.261) were not significantly different between SR and AF groups.

Two groups showed no significant differences considering pre-ECV medical history including medications and systemic diseases (Table 1). Echocardiographic findings of 2 groups are shown in Table 2 (before ECV). The initial LAVI of SR group was 42.21 ± 12.4 ml/m^2^ and AF group was 96.08 ± 52.21 mL/m^2^, the initial LAVI was significantly different between 2 groups (*P* = 0.000)

**Table 1:** The clinical data of two groups (SR and AF)

	SR	AF	P value
HT	25 (49.0%)	8 (15.7%)	0.871
CAD	4 (7.8%)	2 (3.9%)	0.547
CHF	3 (5.9%)	1 (2.0%)	0.942
DM	8 (15.7%)	0 (0%)	0.088
Smoking	7	0	0.114
AFH	6 (11.8%)	3 (5.9%)	0.445
β-Blocker	30 (58.8%)	10 (19.6%)	0.637
CCB	4 (7.8%)	2 (3.9%)	0.547
ACE/ARB	22 (43.1%)	6 (11.8%)	0.583

Data are presented as number (percentage). Chi-square tests were performed. P-value less than 0.05 is significant (*). ACE/ARB: angiotensin converting enzyme inhibitor/angiotensin II receptor blocker, AFH: atrial fibrillation history, CAD: coronary artery disease, CCB: calcium channel blocker, CHF: congestive heart failure, DM: diabetes mellitus, HT: hypertension‏.

**Table 2:** Echocardiographic findings of two groups (SR and AF) before ECV

	SR	AF	P-value
EF (%)	48.9±1.67	49.00±2.46	0.696
LVDD (cm)	4.70±0.14	4.90±0.26	0.284
LVSD (cm)	3.31±0.12	3.84±0.44	0.211
LAVI (ml/m^2^)	43.4±1.9	96.1±15.0	0.000*

Data are presented as mean ‏± standard deviation. Mann-Whitney U-test tests were performed. *P* value less than 0.05 is significant (*). EF: ejection fraction, LAVI: left atrial volume index, LVDD: left ventricular diastolic diameter, LVSD - left ventricular systolic diameter.

The LAVI of SR group decreased significantly (5.69 ± 0.74 mL/m^2^) after 3 months, LAVI decreased from 42.21 ± 12.4 mL/m^2^ to 37.51 ± 10.52 mL/m^2^ (*P* = 0.000). The cut-off point of LAVI value in predicting the maintenance of SR was 55 mL/m^2^.

In the multiple logistic regression analysis carried out after the formation of the model based on the parameters related to AF recurrence, only the relationship with LAVI was observed to be prevalent. Each 1 mL/m^2^ increase in the LAVI was found to be related with a 15% increase in the risk for the recurrence of the AF independently from the other parameters (*P* < 0.001). The results of the regression analysis where the related factors of AF recurrence are evaluated are presented in Table 3.

**Table 3:** Predictors of AF recurrence: The multiple logistic regression analysis Results

**Parameters**	**β**	**OR**	**95% CI**		**P value**
			**Lower**	**Upper**	
Age	0.012	1.012	0.869	1.178	0.270
Sex	1.023	2.781	0.109	70.696	0.247
BMI	0.384	1.468	1.005	2.146	0.35
EF (%)	3.342	28.287	0.000	0.000	0.209
LVDD (cm)	-11.778	0.000	0.000	75.420	0.325
LVSD (cm)	11.402	855225.7	0.011	7.138E1	0.812
LAVI (ml/m^2^)	0.143	1.154	1.053	1.264	<0.001

AF, atrial fibrillation; BMI, body mass index; CI, confidence interval; OR odds ratio. EF, ejection fraction; LAVI, left atrial volume index; LVDD, left ventricular diastolic diameter; LVSD, left ventricular systolic diameter.

**Figure 1:**
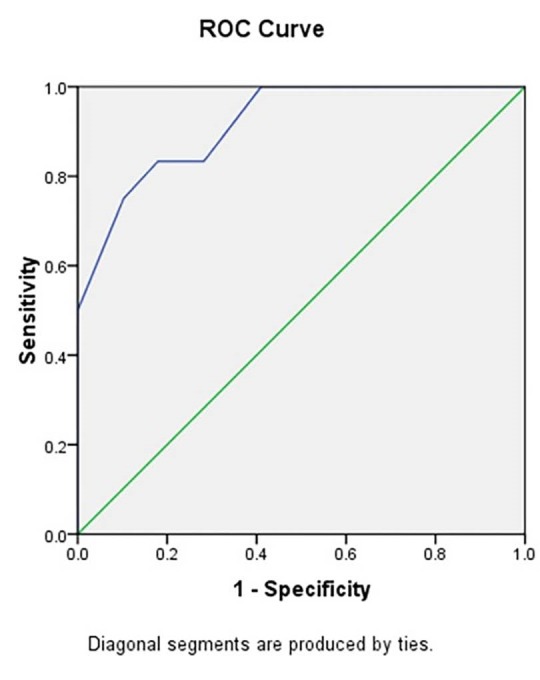
ROC of LAVI as a predictor of maintenance of SR after ECV.

ROC curve are illustrated in Figure 1 to evaluate LAVI as a predictor of maintenance of SR after ECV. The cut-off point of LAVI value in predicting the maintenance of SR was 55 mL/m^2^ (sensitivity: 75.0%, specificity: 89.7%). The LAVI of SR group decreased significantly (5.69 ± 0.74 mL/m^2^) after 3 months ( *P* = 0.000)

## Discussion

In our study, 76.5% of patients could maintain SR three months after ECV which is in total agreement with previous studies.^2,5,9,15,16^ Several studies have been focusing on the predictors of AF recurrence after conversion.^2,5,9,16-18^ According to the demographic data of present study including age and sex, patients did not show any significant difference. The BMI did not also affect AF recurrence significantly. This result is similar to the findings of Frick et al,^2^ Akdemir et al,^13^ and Osmanagic et al^18^ but it is opposed to Blich and Edoute’s.^19^ None of the co-morbid conditions and systemic diseases influenced significantly the recurrence rate of AF in our study. Previous studies have also not reported significant relationship in most conditions,^2,5,13,18^ However some of them mentioned hypertension,^2,17^ diabetes mellitus^17^ and history of previous AF cardioversion ^17^ as influencing factors. In present study, according to many earlier investigations,^13,18^ no significant difference was found in the recurrence of AF regarding pre-ECV medications, although some medications such as beta-blockers have been reported with possible effect on the recurrence.^2,17^

An increase in left atrial size is known to be associated with cardiovascular diseases. The effect of left atrial size on the recurrence of AF has been reported previously and many parameters (readily by anteroposterior diameter) have been suggested in earlier studies to evaluate it.^9,12,15,20,21^ Measurement of anteroposterior linear left atrium dimension by M-mode echocardiography is easy, but not reliable, since the left atrium is not uniformly spherical and anteriorly constrained by the sternum and aortic root and posteriorly by the relatively rigid tracheal bifurcation and spine. Therefore enlargement often takes place in the superior-inferior or mediolateral axis. Thus this unidimensional measurement cannot reflects the exact complexity of changes.^13,22^

The size of left atrium could be measured more accurately by the left atrial volume by two-dimensional echocardiography in comparison to the reference standards such as magnetic resonance imaging and three-dimensional echocardiography.^23-25^ The computation of left atrial volume has been described by two-dimensional echocardiography previously.^26,27^ The LAVI has been applied to investigate cardiovascular conditions by recent studies increasingly.^28-31^

A few numbers of investigations have studied the relationship between the LAVI and the AF recurrence. Wang stated that the LAVI is higher in the patients with atrial fibrillation recurrence after conversion.^12^ Kim et al and then Kataoka et al declared that the LAVI, as opposed to the conventional left atrial dimension, was supposed to be an important predictor of successful SR restoration after the maze operation.^30,32^ Similar statement was suggested by Lee et al for occurrence of atrial fibrillation after ablation of typical atrial flutter.^33^ Marchese et al and also Akdemir et al concluded that larger LAVI before ECV, as a more accurate measure than left atrium diameter, was strongly associated with higher risks of AF recurrence.^13,17^

Among our echocardiographic data, LAVI is the only measurement which predicts the recurrence of AF. Ejection fraction (EF), left ventricular diastolic diameter (LVDD), and left ventricular systolic diameter (LVSD) were not significantly different between SR and AF groups. These are in total agreement with the results of Akdemir et al and Marchese et al. The LAVI values of SR group in Marchese et al (31.4 ±4.6 mL/m^2^) and Akdemir et al (35.3 ±11.5 mL/m^2^) studies were close to our results (43.4 ± 1.9 mL/m^2^) and also the values of normal patients (34 mL/m^2^).^34^ However, the patients with the recurrence of AF had larger LAVI in our study. (96.1 ±15.0 mL/m^2^ in our study comparing to 39.7 ±8.4 mL/m^2^ in Marchese et al and 53.1±10.1 mL/m^2^ in Akdemir et al studies).^13,17^

The cutoff point of LAVI value in predicting the maintenance of SR was 55 mL/m^2^ (sensitivity: 75.0%, specificity: 89.7%) in our study which was higher than 40 mL/m^2^ (sensitivity: 38%, specificity: 96%) in Marchese et al and 36 mL/m^2^ (sensitivity: 100%, specificity: 82.5%) in Akdemir et al studies.^13,17^ The higher LAVI values of AF group in our study may be associated with the longer duration of AF before the ECV, the difference in the measurement method of LAVI, the characteristics of the patient population and/or the duration of follow-up after ECV.

Another founding of present study was a decrease in LAVI after 3-month maintenance of SR. This result besides the aforementioned role of LAVI in prediction of AF recurrence could lead us to the concept of left atrial remodeling. Left atrial remodeling is a time-dependent adaptation of cardiac myocytes to maintain homeostasis over external stressors. The high rates of cell depolarization and volume/pressure overload in AF could be a major stressor. Increased volume/pressure overload gives rise to dilatation and stretch of the atrium. Atrial remodeling finally could results in many structural, functional, electrical, metabolic, and neurohormonal consequences which are mostly reversible (in cellular level, the apoptosis and fibrosis are usually irreversible).^17,35^

The atrial size and specifically LAVI could reflect the macroscopic aspect of this remodeling. Determining of an irreversible threshold for atrial remodeling in AF may be difficult but achievable. This study presents cutoff point of LAVI enlargement which could be a used as a clinical value for distinguishing AF recurrence based on the undermining microscopic irreversible changes.

The present study forecasts that LAVI is a powerful indicator of the recurrence of AF after ECV. The LAVI measurement could be a useful method in the selection of the patients with AF for ECV. Despite the evidence from our study and also previously mentioned studies, the latest guidelines for AF management have not included the LAVI in echocardiographic examination yet.

## Study limitations

The main limitation of this study is the relatively small study population which may not devaluate the results of this investigation due to a statistically well-controlled sampling and analysis. Another limitation would be that the continuous event recorder didn’t use during follow-up and missing of transient asymptomatic episodes of AF was possible.

## Ethical Approval

The study protocol was approved by the ethics committee of Tabriz University of Medical Sciences.

## Competing interests

Authors declare no conflict of interest in this study.

## Acknowledgements

We would like to thank Dr. Sohrab Pourkhameneh for his valuable assistance. Cardiovascular Research Center of Tabriz University of Medical Sciences supported the grant of present research.
